# Genome structural dynamics: insights from Gaussian network analysis of Hi-C data

**DOI:** 10.1093/bfgp/elae014

**Published:** 2024-04-22

**Authors:** Anupam Banerjee, She Zhang, Ivet Bahar

**Affiliations:** Laufer Center for Physical & Quantitative Biology, Stony Brook University, NY 11794, USA; OpenEye, Cadence Molecular Sciences, Santa Fe, NM 87508, USA; Laufer Center for Physical & Quantitative Biology, Stony Brook University, NY 11794, USA; Department of Biochemistry and Cell Biology, Renaissance School of Medicine, Stony Brook University, NY 11794, USA

**Keywords:** chromatin dynamics, 4D Genomics, Gaussian network model (GNM), Hi-C, genome hierarchical organization, gene expression regulation

## Abstract

Characterization of the spatiotemporal properties of the chromatin is essential to gaining insights into the physical bases of gene co-expression, transcriptional regulation and epigenetic modifications. The Gaussian network model (GNM) has proven in recent work to serve as a useful tool for modeling chromatin structural dynamics, using as input high-throughput chromosome conformation capture data. We focus here on the exploration of the collective dynamics of chromosomal structures at hierarchical levels of resolution, from single gene loci to topologically associating domains or entire chromosomes. The GNM permits us to identify long-range interactions between gene loci, shedding light on the role of cross-correlations between distal regions of the chromosomes in regulating gene expression. Notably, GNM analysis performed across diverse cell lines highlights the conservation of the global/cooperative movements of the chromatin across different types of cells. Variations driven by localized couplings between genomic loci, on the other hand, underlie cell differentiation, underscoring the significance of the four-dimensional properties of the genome in defining cellular identity. Finally, we demonstrate the close relation between the cell type–dependent mobility profiles of gene loci and their gene expression patterns, providing a clear demonstration of the role of chromosomal 4D features in defining cell-specific differential expression of genes.

## INTRODUCTION

The spatial organization of the eukaryotic genome and the time-dependent interactions of genomic elements, shortly termed the four-dimensional (4D) properties of the genome, hold the key to unraveling the intricate mechanisms of transcriptional regulation and epigenetic modifications [1–5]. They also underlie developmental processes that guide cell growth and differentiation [6]. Aberrations in genome organization can lead to a wide range of disease conditions, as disruptions in spatial arrangement can perturb normal gene regulation [7]. Furthermore, changes in spatial organization can drive adaptations that shape the evolutionary trajectory of species over time [8].

Advances in the characterization of spatial connections between gene loci using methods like fluorescent *in situ* hybridization (FISH) or Chromosome Conformation Capture (3C) have played a major role in gaining insights into the genome three-dimensional (3D) structure [9]. With advances in next-generation sequencing (NGS), 3C methods evolved into high-throughput chromosome conformation capture (Hi-C), allowing for genome-wide analysis of chromosomal interactions and providing a more complete view of the organization of the chromatin in humans and other species [10–13]. Insights from Hi-C and computational methods developed in parallel have yielded a deeper understanding of the chromosomal organization, revealing a hierarchical architecture.

The hierarchical architecture is a fundamental aspect of 3D genome organization [6, 14]. At its most basic level, DNA is tightly wound around histones into nucleosomes. Nucleosomes are compacted into chromatin fibers, which, in turn, are organized into chromatin loops and topologically associating domains (TADs), i.e. chromosomal territories with gene loci interacting among themselves within the territory [10]. TADs, in turn, are grouped into compartments that can be transcriptionally active (A) or repressive (B) [11]. This hierarchical organization thus allows for the spatial segregation of active and inactive regions of the genome. Moreover, the looping of chromatin fibers, facilitated by protein complexes like CTCF and cohesin, plays a crucial role in shaping the higher-order architecture, allowing enhancers and promoters to come into interaction range and regulate gene expression [15]. Overall, the hierarchical architecture of the chromatin is essential to the orderly orchestration of genomic processes and gene regulation within the cell.

Hi-C data analysis methodologies can be grouped into four categories: template-based, structural, statistical and learning-based. Template-based methods use known image patterns for interpretation; structural methods aim to construct 3D models of chromatin organization by mapping the data on interactions to matrix or graph representations. Statistical techniques detect significant interactions and compartments, while learning-based methods employ machine learning (ML) for prediction and classification. Here we focus on a structure- or, more precisely, 3D-contact topology-based computational method, Gaussian network model (GNM) [16], rooted in the statistical mechanics of macromolecular systems [17–19]. The GNM permits us to learn how the genome topology defines the equilibrium fluctuations of gene loci, and how these fluctuations then potentially influence, if not determine, gene expression and regulation events.

### Significant advances have been made in recent years in computer modeling of chromatin 3D organization

On the computational side, the discovery of the plaid pattern in Hi-C maps, distinguishing A and B compartments, was initially achieved through principal component analysis (PCA). Since then, PCA has become a standard method for compartment identification, with various tools like Juicer [20], HOMER [21], HiCdat [22], POSSUMM [23] and dcHiC [24] employing different implementations. For instance, POSSUMM accelerates eigenvector decomposition via the power method, while dcHiC implements a parallelized partial singular value decomposition (SVD) for efficient compartment analysis. Alternative methods like Cscore Tool [25] and Calder [26] have been introduced, offering statistical-based and clustering approaches for compartment analysis.

TAD detection has advanced with a variety of algorithms, while research on compartments and subcompartments has been limited. Initially, TAD identification looked for consecutive diagonal squares indicative of frequent interactions on gene–loci contacts heatmaps, but recent methods evolved to predict hierarchical structures. The usual approach is boundary detection and use optimization algorithms, with examples like Armatus [27], Insulation Score [28], Arrowhead [12] and HOMER’s findTADsAndLoops [29]. Learning-based pattern recognition includes hierarchical clustering (e.g. Constrained HAC [30] and TADpole [31]) and partitional algorithms (e.g. ClusterTAD [32]). Statistical pattern recognition techniques, on the other hand, employ z-scores, BIC-penalized likelihood (e.g. TADbit [33]) and Poisson distributions (e.g. chromoR [34]). Tailor-made models like TADTree [35] and PSYCHIC [36] produce overlapping or nested TADs.

Hi-C data are typically population-averaged, representing an ensemble average; hence, not all contacts are simultaneously satisfied. The data provide a probabilistic description of contacts that may be used to construct optimal structural models. A wide range of computational methods have been developed for reconstructing the genome 3D structure from Hi-C data, including distance- and contact-based methods, as recently reviewed [37, 38]. Notable studies include the generation of ensembles of chromatin structures by entropy maximization principle, by Shi and Thirumalai [39] and Lin *et al.* [40]. Seminal work by Onuchic and coworkers also demonstrated the utility of using epigenetic data from Chromatin Interaction Analysis by Paired-End Tag Sequencing (ChIA-PET) experiments in a maximum-entropy-based neural network model for predicting chromosomal architecture [41, 42], now implemented in the software PyMEGABASE [43].

We focus here, not on the 3D reconstruction of the chromatin using Hi-C data, but on the assessment of equilibrium/fluctuation dynamics of the chromatin, as defined by the gene loci contact topology. These properties are computed with the help of GNM, a simple elastic network model (ENM). Notably, the GNM does not require explicit knowledge of 3D structure, but just 3D contact topology. As such, it does not rely on structural models (or ensemble of models) that optimally comply with Hi-C data. Instead, it uses the original data on loci–loci contacts, thus avoiding any uncertainties that may originate from approximate modeling of the genome 3D structure. The information GNM yields on equilibrium fluctuations in the spatial positions of gene loci as well as on their cross-correlations is directly inferred from the experimentally measured strengths and/or probabilities of inter-loci contacts. It further allows to dissect the spectrum of fluctuations into normal modes and extract the most dominant/cooperative events, which often relate to function. As presented below, the elucidation of this type of 4D behavior helps us gain insights into the physical basis of observed gene expression or regulation data. It also provides a clear description of the hierarchical structure of the chromatin, the coordinated biological activity of (sequentially) distant genomic loci and the cell type specificity of chromatin mobility.

### ENMs provide a rigorous description of spatial correlations defined by contact topology

ENMs have proven in the last two decades to lend themselves to highly versatile methods for predicting the signature dynamics and allosteric responses of biomolecular systems [44–48]. They have been more recently extended to the structure and dynamics of the chromatin as inferred from Hi-C data [49], as well as the identification of effectors and sensors of information flow within cellular gene networks [50]. Their use in tandem with Hi-C data helped gain insights into the cell-type dependency of gene loci fluctuations and their correlation with gene expression patterns [51].

ENMs approximate complex systems as networks, the nodes of which are connected by springs (governed by harmonic potentials) such that the nodes are subject to Gaussian spatial fluctuations. This representation presents the advantage of yielding a unique analytical solution for the equilibrium/fluctuation dynamics of the system, which, upon spectral (normal mode) decomposition, permits us to extract global couplings between nodes [52]. Global motions refer to cooperative movements of clusters of nodes (e.g. domains in proteins, or TADs or compartments in the chromatin) at the low frequency end of the spectrum. Higher frequency modes, on the other hand, point to spots of energy localization, which are usually evolutionarily conserved and define the ‘core’ interactions that underlie the stability of structure. Thus, ENMs help us gain a mechanistic understanding of relaxational movements or intrinsic dynamics accessible to the structure while maintaining the topology, on the one hand, and restrictions in accessible space dictated by the overall topology, on the other.

ENMs are scalable and could be used for events at multiple scales. Techniques for predicting structural patterns from Hi-C data typically focus on relatively short-range interactions (within 1–2 million base pairs), or occasionally up to 10 million bps. However, ENMs can expand the scope of predicted couplings to encompass gene loci that are tens of millions of base pairs apart, including inter-chromosomal couplings [49]. These long-range correlations could arise from physical proximity in 3D space or from *trans*-regulatory effects, resembling the allosteric effects seen in biomolecular structures. ENMs quantify the accessibility of and cross-correlations between genomic loci at different scales, while simply using the shape and frequency of the mode spectra to differentiate across cell types.

### GNM provides a mathematically exact description of couplings within the genome

Currently, there is a need for a comprehensive framework capable of automatically recognizing from Hi-C data the hierarchical levels of couplings within the genome. These couplings occur at different scales [53], from individual genes (gene–gene co-expression) to large domains such as TADs and compartments, including entire chromosomes. ENM-based spectral decomposition of chromatin dynamics provides such a hierarchical description as will be elaborated below.

In this review, we illustrate the utility of ENMs, particularly the GNM [16, 54], for studying chromatin dynamics, when analyzing data generated from Hi-C experiments [49, 51]. The GNM, inspired by the Rouse model of polymer physics [17], solely considers the inter-node contact topology of biological macromolecules as an elastic network of masses and springs to characterize its equilibrium dynamics. Hence, it is directly applicable to the inter-loci contact topology observed in Hi-C matrices. GNM offers four main advantages: (i) it is mathematically rigorous and yields an exact solution for each network architecture; (ii) it is grounded in well-established principles of physical sciences and solid-state physics applicable to intrinsically elastic systems such as DNA; (iii) it is computationally efficient and can be performed at hierarchical levels of resolution; and (iv) it yields concrete, quantitative data amenable to comparison with experiments and to intuitive interpretations. It is agnostic to the atomic details of the gene loci and does not take account of the specific atomic interactions while determining the equilibrium dynamics of chromatins. In this respect, it is purely geometry-based and reflects entropically driven behavior.

**Figure 1:**
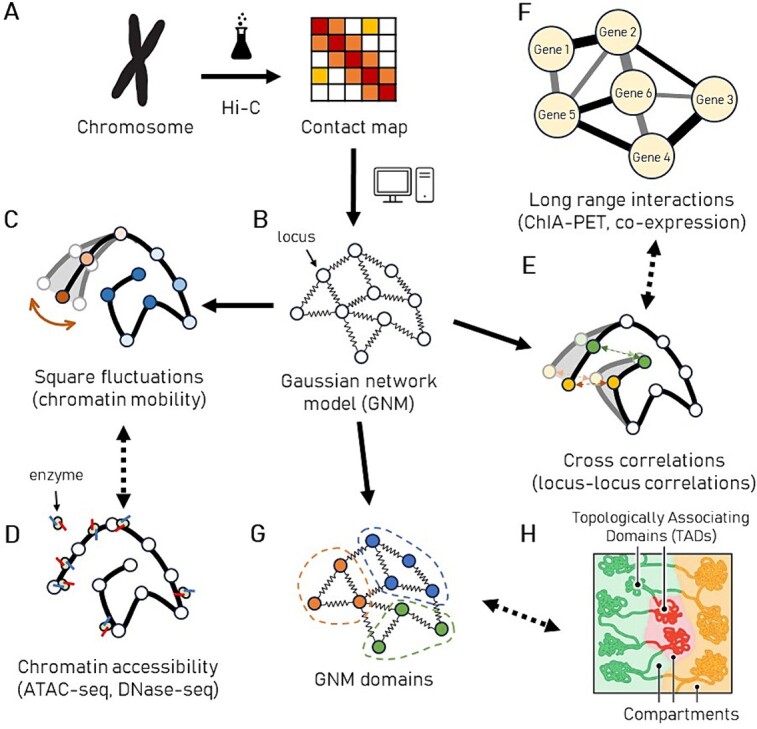
Schematic representation of relationships between the GNM predictions and various genome-scale experimental data. (**A**) The Hi-C experiments capture the topology of contacts in the chromatin by performing a high-throughput ligation assay on chromosomes. The topology is expressed as a *n* × *n* contact map for *n* gene loci. Each locus is of several kilo- to mega-base pairs, depending on the resolution of the maps. (**B**) The GNM is an elastic network representation, built from the contact map where each node represents a locus, and springs represent the contact strengths between pairs of loci. (**C**) The square fluctuations near mean positions can be calculated for each locus using the GNM. Loci that are more exposed are expected to be more flexible/mobile. The diagram shows the sequence of loci (solid black line); the nodes being colored by mobility (orange: high, blue: low). Predicted chromatin mobility strongly correlates with (**D**) chromatin accessibility measured by either ATAC-seq or DNase-seq. (**E**) Pseudo-inversion of the GNM contact map yields an *n* × *n* cross-correlation map. Each entry in the map represents the cross-correlation between loci pairs (numbered along the axes). (**F**) Cross-correlation maps are compared to, and validated by, experimental data on couplings between loci or gene pairs, including ChIA-PET data or gene co-expression network. (**G**) Structural domains identified by GNM spectral analysis can be compared with chromosomal structural such as compartments (in different colors) and TADs (blobs) illustrated in (**H**). (**H**) is adapted from Rao *et al.* [12].

A schematic illustration of the application of GNM to Hi-C data analysis and its extraction of properties relevant to various genome-scale data is shown in Figure 1. GNM-based analysis facilitates the assessment of various genome-wide properties, including loci mean-square fluctuations (MSFs), regulatory interactions and the identification of TADs and compartments, all in alignment with experimental results. The framework also reveals spatially coupled regions called cross-correlated distal domains (CCDDs) [49]. Notably, these predictions properties show good accord with experimental data in support of the utility of GNM for establishing the physical bases of gene regulation.

### Underpinnings of GNM methodology adapted to exploring chromatin 4D behavior

The Hi-C process involves fixing cells with formaldehyde to preserve their spatial organization, fragmenting their chromatin (DNA and associated proteins) with a restriction enzyme and labeling the ends with biotin, and then ligating proximate (cross-linked) DNA fragments together. After biotin pull-down experiments, the extracted DNA segments are sequenced with NGS techniques [12, 55]. The data generated are used to construct a Hi-C contact matrix, portraying the frequency/strength of pairwise interactions between genomic loci across the entire genome.

The GNM has been used to quantify the equilibrium dynamics for a wide variety of biomolecular systems from polymer networks [18, 19] to proteins/DNA molecules [16, 47, 54] or ensembles of sequentially heterogeneous proteins that belong to the same family [45], and recently to chromatin [49, 51]. The only ingredient of the theory is the Hi-C contact matrix. This is a symmetrical square matrix, with rows and columns representing the sequential gene loci along the DNA, and the entries are the interaction frequencies. It may be viewed as a large matrix composed of submatrices, the diagonal submatrices representing the interactions between gene loci within the same chromosome, and the off-diagonal matrices, between the chromosomes. The data can be generated (and represented) at various resolutions, ranging from high resolution that captures fine-scale interactions (e.g. 1 kilobase (kb) resolution, i.e. each locus is composed of 1000 base pairs) to lower resolution offering a more condensed view of interactions (e.g. 100 or 1 Mb resolution) [37, 56, 57].

The adaptation of the GNM to analyzing chromatin fluctuation dynamics requires the identification of nodes by gene loci (or groups of them, depending on the resolution of the adopted model), and the construction of a connectivity/Kirchhoff matrix, **Γ**, describing the topology of contacts across the chromatin. The *ij^th^* element Γ_ij_ of **Γ** is defined as


(1) \begin{equation*} {\Gamma}_{ij}=\left\{\begin{array}{@{}c}-{w}_{ij}\ \mathrm{if}\ i\ne j\\{}\sum_{i,i\ne j}{w}_{ij}\ \mathrm{if}\ i=j\end{array}\right. \end{equation*}

where ${w}_{ij}$ is the number (or frequency/strength) of contacts observed in Hi-C experiments between loci $i$ and $j$. The diagonal element ${\mathrm{w}}_{ii}$ is the negative sum of non-diagonal elements in the *i^th^* row (or column), thus providing a measure of the total number/strength of contacts experienced by a given node (or gene loci). Elastic springs of uniform force constant, γ, map to Gaussian distribution of distances between nodes, centered on their average values. The key factor is the contact topology, which aligns well with an ensemble of conformers maintaining node positions. The use of population-averaged Hi-C data naturally assigns higher effective spring constants to pairs of loci that exhibit relatively more stable/frequent interactions.

The cross-correlation between the spatial displacements $\Delta \boldsymbol{r}_i\ \mathrm{and}\ \Delta \boldsymbol{r}_j$ of the respective loci $i$ and $j$ is evaluated from the pseudoinverse of **Γ** and expressed as the *ij^th^* element of the cross-correlation matrix **C**, as


(2) \begin{equation*} {C}_{ij}=\left\langle \Delta \boldsymbol{r}_i.\Delta \boldsymbol{r}_j\right\rangle \propto{\left[\boldsymbol{\Gamma}^{-1}\right]}_{ij}=\sum_{k=1}^{n-1}\frac{1}{\lambda_k}{\left[\boldsymbol{u}_k\boldsymbol{u}_k^T\right]}_{ij} \end{equation*}

Here, the summation is performed over all the *n-1* non-zero modes of collective motion accessible to the *n* nodes. Each mode *k* is characterized by a shape (elements of the *n*-dimensional eigenvector $\boldsymbol{u}_k$ of **Γ**) representing the relative size of the nodes in that particular mode, and a frequency, scaling with inverse square-root of the *k^th^* eigenvalue *λ_k_* of **Γ**. The modes are ordered in increasing frequency from *mode 1* (lowest frequency) to *n*-1 (highest frequency). The term $\left(1/{\lambda}_k\right)\left[\boldsymbol{u}_k\boldsymbol{u}_k^T\right]$ is an *n* x *n* matrix that represents the contribution of the *k^th^* mode to **C**. The *i*^th^ diagonal element **C***_ii_* represents the mean-square fluctuation (MSF) ($\left\langle \Delta{r_i}^2\right\rangle$) of the *i*^th^ locus around its mean position. Note that the eigenvalues are normalized such that the trace $tr(\boldsymbol{u}_k\boldsymbol{u}_k^T$) is equal to 1. The term ${\left(1/{\lambda}_k\right)}^{1/2}$ thus uniformly scales the size of motion undergone by the nodes, such that lower-frequency modes drive larger amplitude motions that usually embody a large portion (if not all) of the structure, hence their qualification as global modes. In other words, the front term $\left(1/{\lambda}_k\right)$ serves as a statistical weight for the contribution of mode $k$ to $\left\langle \Delta \boldsymbol{r}_{i}^2\right\rangle$ or $\left\langle \Delta \boldsymbol{r}_i.\Delta \boldsymbol{r}_j\right\rangle$.

The suitability of the GNM for analyzing Hi-C data is underscored by several key factors. First, Hi-C data involves large-scale genomic information, with human chromosomes spanning millions of base pairs and resulting in thousands of bins per chromosome when binned at 5 kb resolution. GNM's scalability allows it to effectively characterize complex molecular systems with tens of thousands of nodes, making it well suited for efficiently handling and analyzing high-resolution intrachromosomal contact maps generated by Hi-C experiments.

Second, the collective motions in the low-frequency regime predicted by the GNM are robust to imprecision in coordinates (spatial positions of nodes) or inherent noise in the data. Unlike high-resolution structural techniques such as X-ray crystallography and NMR, Hi-C data often includes noise and unmapped regions, leading to lower precision. GNM global modes are insensitive to data precision and resolution at a local level, as demonstrated in earlier work [49], since the collective motions result from the overall contact topology rather than detailed spatial coordinates.

Third, the inherent flexibility of GNM aligns with the dynamic nature of chromatin. Chromatin structures can vary between cells and contexts, exhibiting less rigid organization compared to molecular structures. Single-cell Hi-C experiments have revealed significant variability in chromosome structure between cells while maintaining consistent domain organization at larger scales. The GNM's ability to assess dynamic features at a probabilistic level is compatible with the ensemble nature of the data.

Finally, the Gaussian distribution of fluctuations holds true as the network size increases due to the central limit theorem; as such GNM is ideally suited for investigating the collective dynamics of ‘large’ systems composed of large numbers of nodes like the chromatin.

GNM yields a unique analytical solution to the equilibrium dynamics of the chromatin. The evaluation of properties associated with equilibrium dynamics, for example, the MSFs in the spatial positions of the genomic loci and their cross-correlations, helps us make inferences on mechanisms of genome regulation intrinsically defined by the 3D contact topology. The observed correlations between GNM-predicted MSFs of gene loci and gene loci accessibility data from chromatin accessibility experiments (see below) calls for further GNM analyses to potentially identify previously unknown or unexplained means of genome regulation.

Recent studies have demonstrated the efficacy of Laplacian-based graph segmentation in the identification of topological domains from Hi-C data [58–62]. It is worth noting that, in contrast to most methods that normalize the diagonal elements of the Laplacian matrix post-construction, there is no such normalization in the construction of the GNM Kirchhoff matrix. The latter exactly yields the sum of harmonic potentials that represents the overall energy of the network when substituted in 


(3) \begin{equation*} {\text{E}} =1/2\ \gamma \Delta{\textbf{R}}^{\text{T}} \boldsymbol{\Gamma} \Delta{\textbf{R}} \end{equation*}

where Δ**R** is the *n*-dimensional array of the displacements of the *n* gene loci $\Delta{r}_i,1\le i\le n$ Furthermore, the diagonal elements are physically meaningful, representing the packing density of the gene loci.

### Mean-square fluctuations of gene loci correlate with chromatin accessibility

As shown earlier in an application to H/D exchange data, GNM-predicted MSFs of nodes are purely entropic in nature and provide a good measure of solvent accessibility [63]. The GNM MSFs predicted for gene loci also yielded strong correlation with chromatin accessibility experiments [49], DNase-seq and ATAC-seq, as illustrated in previous work for two distinct cell lines: GM12878 and IMR90 [64, 65]. DNase-seq involves enzymatic digestion of DNA and ATAC-seq using a hyperactive enzyme to identify open chromatin regions.

**Figure 2:**
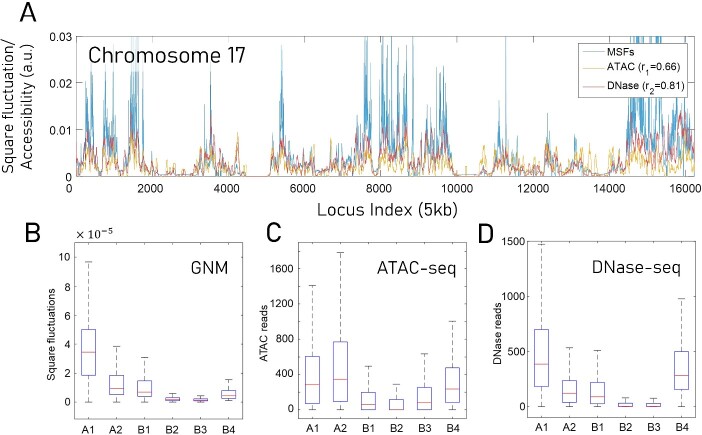
GNM-predicted mobilities of chromosomal loci show good agreement with experimentally measured chromatin accessibility. (**A**) Square fluctuations of loci (chromatin mobility) obtained from GNM analysis of the equilibrium dynamics of chromosomes 17 for human lymphoblastoid cell line (GM12878), are compared to the DNA accessibilities probed by ATAC-seq and DNA-seq experiments. GNM results represent the average over 500 lowest frequency modes evaluated using a 5 kb-resolution Hi-C map. *r_1_* is the Spearman correlations between GNM predictions and ATAC-seq experiments; and *r*_2_ is that between GNM and DNase-seq. All data are normalized for visual comparison. (**B**) GNM-predicted loci mobility for different types of subcompartments, within the A and B compartments. The box plot shows the distribution of the square fluctuations of loci located in each compartment type. (**C**) ATAC-seq and (**D**) DNase-seq measured chromatin accessibility for the same subcompartments. The box plots show the read concentration, i.e. number of reads per locus, of loci located in each compartment type. Results in (**B**–**D**) are obtained using data for all chromosomes of GM12878.

Comparison of GNM-predicted and experimental DNase-seq data revealed a Spearman correlation of 0.80 ± 0.04 averaged across all 23 chromosomes of GM12878 cells, while the correlation between GNM MSFs and ATAC-seq data was 0.55 ± 0.11. Notably, the experimental DNAse-seq and ATAC-seq data themselves exhibited a correlation of 0.74 ± 0.09. These results suggests that DNase-seq experiments show higher agreement (than ATAC-seq) with the computational predictions. Figure 2A illustrates the comparison between theory and experiments for chromosome 17 in GM12878 cells. We selected chromosome 17 for illustrative purposes. Similar correlations have been shown for all other chromosomes in earlier work [49].

Further comparison with experimental data on IMR90 cells consistently demonstrated even stronger correlations between GNM predictions and experimental data, yielding respective Spearman correlations of 0.82 ± 0.03 with DNase-seq and 0.63 ± 0.08 with ATAC-seq. These findings support the utility of GNM as a predictive tool for exploring the exposure of gene loci to the environment. It is worth noting that the analysis maintained its robustness when repeated at different resolutions, from 5 to 50 kb per locus.

Compartments A and B, initially identified in early Hi-C studies [11], represent open and closed chromatin regions, respectively, based on the PCA of Hi-C contact matrices. Subsequent advances in Hi-C techniques revealed six subcompartments (A1, A2, B1, B2, B3, B4) within these compartments [12]. Figure 2B illustrates GNM-predicted MSFs for these subcompartments. The results are statistically significant (*P*-value <0.01) and in accord with the higher mobility and accessibility of open chromatin regions (compartment A) compared to closed chromatin (compartment B). Further examination of subcompartments reveals heterogeneous chromatin properties within the same compartment. Specifically, A2 displays variations similar to B1, which is in agreement with DNase-seq data (Figure 2D), but not ATAC-seq (Figure 2C). This observation aligns with A2's association with H3K9me3, a marker for heterochromatin [12]. Among compartment B subtypes, B2 and B3 were the most rigid, again consistent with DNase-seq data and with the finding that they are enriched at nucleolus-associated domains or lamina-associated domains. B1 and B4, on the other hand, consistently showed relatively enhanced mobilities and accessibility with respect to other B subtypes.

### Characterization of hierarchical levels of organization in the chromatin

Subcompartments detection in Hi-C data lacks standardization. Methods like GaussianHMM [12] identified six subcompartments using clustering algorithms, while SNIPER [66] employed neural networks to categorize A and B compartments into five subcompartments. Calder [26] computed score matrices, using PCA and hierarchical clustering with log-normal distribution for subdomain estimation. dcHiC [24] uses hidden Markov models based on the first PC. Graph-based methods like SCI [67] transform interaction graphs and apply *k*-means clustering for subcompartment prediction. Notably, all methods rely on statistical or learning-based paradigms and use graph representations, lacking template-based or structural pattern recognition approaches.

**Figure 3:**
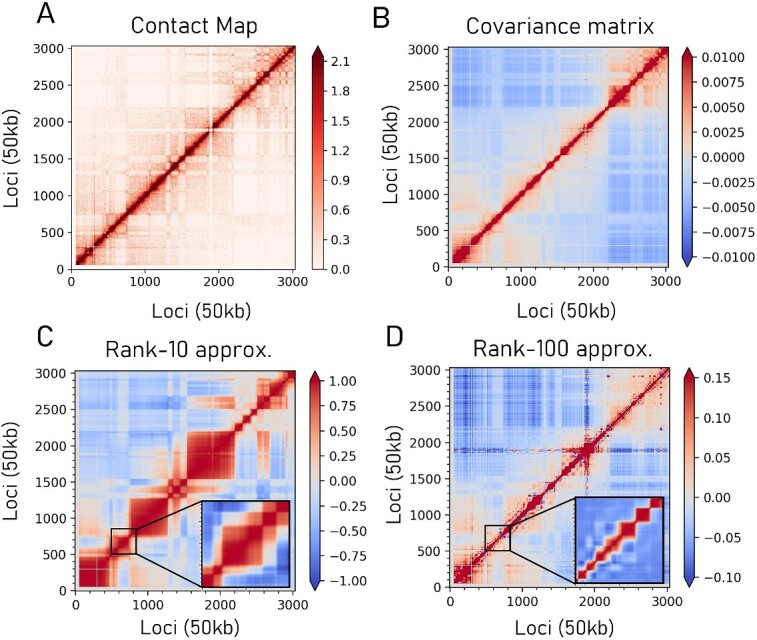
GNM-predicted loci co-mobility at different resolutions. (**A**) Contact map of mouse chromosome 5 (vanilla coverage normalized). (**B**) covariance matrix (cross-correlations) calculated by the GNM using the contact map. (**C**, **D**) Low rank approximation of the covariance matrix shows chromosomal structural features at different scales.

The multiscale nature of GNM spectra allows for exploring hierarchical levels of organization within the chromatin. As mentioned above, GNM low frequency modes capture global dynamics, and higher frequency modes represent localized motions, such that global modes point to large-scale organizations; whereas successive inclusions of higher frequency modes enable the detection of increasingly smaller size clusters. In Figure 3, we present an example gene loci covariance at various resolutions. Figure 3B corresponds to the covariance map from the Hi-C map in Figure 3A, while Figure 3C and D represent the covariance maps obtained with subsets of 10 and 100 (lowest frequency) modes. Cross-correlated gene clusters of various sizes are discerned, the size of the clusters decreasing with increasing number of modes included in evaluating the covariance.

Subsets of GNM modes thus distinguish domains obeying similar dynamics at different time scales. Their correspondence to TADs was examined using the Armatus TAD caller [27]. Optimal parameters resulted in a significantly low variation of information (VI) distance [68] for all 23 chromosomes. Approximately 35 GNM modes were required to capture TAD-like structures, and about 12 GNM modes sufficed for discerning compartment-like structures, illustrating the GNM's robustness to the resolution of the Hi-C matrix [49]. Furthermore, the first 5–20 non-zero modes corresponded fairly well to larger-scale compartments, with optimal VI distances compared to Lieberman–Aiden compartments. This analysis demonstrated the flexibility and generality of the GNM-based spectral decomposition in capturing both TADs and compartments across different scales.

### Distal loci predicted to undergo correlated dynamics exhibit high correlations in their biological activities

In previous work [49], covariance maps were generated to characterize long-range interactions driven by the complete spectrum of modes for the 23 chromosomes within GM12878 cells, represented at 5 kb resolution. Repeating the computations using lower resolution data (50 kb) and fewer modes (500 modes) yielded maps that consistently retained the same features regardless of the granularity of the model. Furthermore, inclusion of a larger number of modes (e.g. >500) would minimally affect the fluctuation behavior of the chromosomes.

Robust couplings between locus pairs separated by megabases were evident in the covariance matrix. Notably, at 5 kb resolution, loci intervals of 200 (which may be viewed as ‘close neighbors’ along the DNA) actually correspond to loci pairs separated by 1 Mb, which would not be coupled if it were not for the compact packing of the genome. A detailed examination of such loci pairs revealed varying levels of cross-correlations, which aligned well with data from ChIA-PET experiments [69]. ChIA-PET experiments identify interactions between loci separated by several hundreds of kb [70]. To validate these findings, we compared GNM-predicted cross-correlations between background pairs separated by the same one-dimensional (sequential) distance on both sides of ChIA-PET pairs along each of the 23 chromosomes. Notably, background pairs exhibited weaker GNM cross-correlations with ChIA-PET pairs compared to those loci pairs predicted to be coupled, yielding *P*-values less than 10^−19^ (two-sided *t*-test), in support of the ability of the GNM to distinguish between coupled and decoupled gene loci.

Furthermore, we observed that the inter-loci cross-correlations did not necessarily scale with the frequency of contacts detected by Hi-C. Instead, a wide range of cross-correlations was noted for a given number of contacts, suggesting that the local number of contacts are not the major determinants of the observed couplings. Instead, the cross-correlations represent global properties defined by the entire network topology, i.e. they are dominated by the collective behavior of the entire structure.

In the context of gene expression correlations from 212 experiments, the GNM covariance map unveiled correlations between distant genomic regions (>10 Mb apart), emphasizing the presence of cross-correlated distal domains (CCDDs). CCDDs, comprised pairs of dynamically linked regions along the chromosome, exhibit relatively high covariance values compared to other regions at similar genomic distances. In contrast to prior methods that concentrated on local chromatin interactions, CCDDs are tens of megabases apart. The covariance matrix effectively captured long-range correlations arising from complex loci–loci contacts. Moreover, genes situated within CCDDs separated by 50 to 100 Mb demonstrated significantly higher co-expression compared to background gene pairs, implying that their distinguishably high cross-correlations have biological significance. This comprehensive exploration underscores the utility of GNM in identifying spatially distant pairs of genomic regions that may be co-expressed.

### Global motions of the genome are highly conserved among different cell types

Cell identity is shaped by lineage-specific gene expression during differentiation [71]. This process is dependent on the accessibility of DNA regions to transcription factors and co-factors, influenced by the genome's spatial organization. Diverse cell types exhibit distinctive chromatin contact patterns associated with their development [72–77]. Advances in Hi-C techniques and computational genomic structural analyses enable exploration of genome-scale differences across cell types. Rao *et al.* [12] identified conserved loop domains (~100 kb) across cells and species. While chromatin domain boundaries tend to remain stable during cell differentiation, substantial changes occur within and between domains [74]. The conservation of CTCF sites, which determine domain boundaries, varies [78]. Recent single-cell studies reveal that larger chromatin structures remain relatively constant, but TADs and loops can vary even within the same cell type [13].

**Figure 4:**
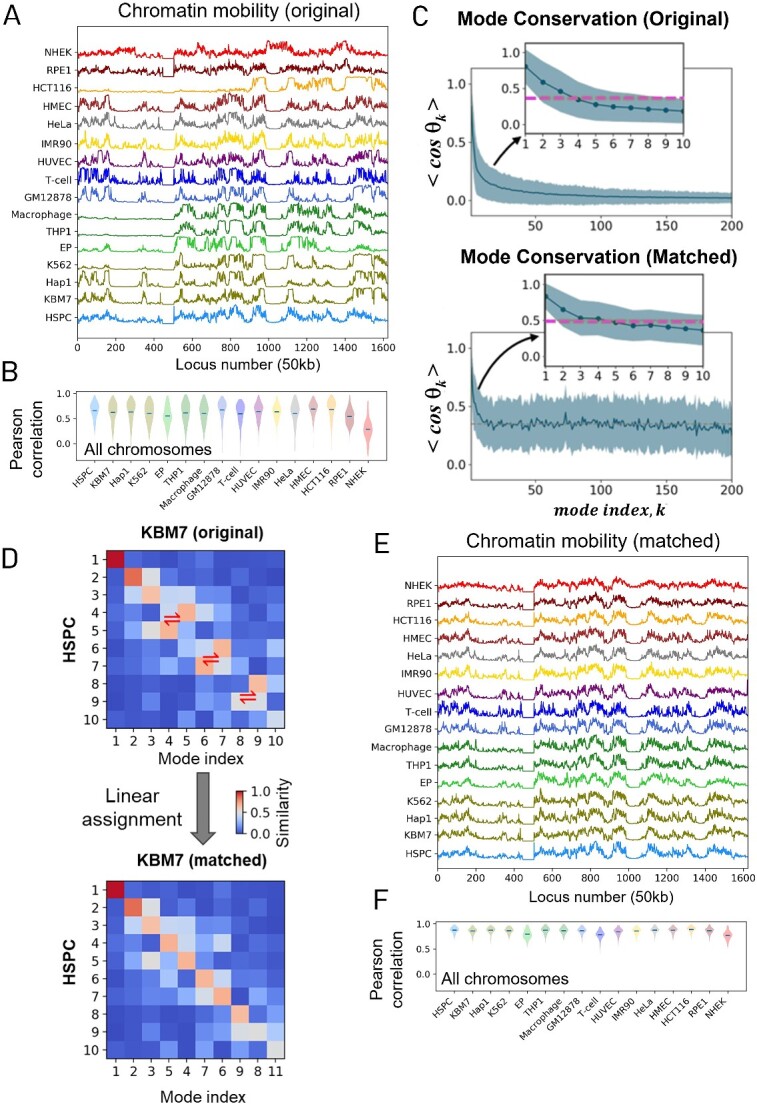
Shared chromatin dynamics across 16 different cell types. (**A**) Mobility (MSF) profiles of gene loci on chromosome 17 computed for 16 different cell types listed along the ordinate, based on 500 GNM modes. (**B**) Pearson correlations between the mobility profile of each cell, and that of all other cells, averaged over the other cells. (**C**) Mode conservation profile. The top panel shows the average pairwise correlation cosine, < *cos* θ*_k_* >, between the *k^th^* collective mode of the chromatin (*n*-dimensional vector ${u}_k$ for a network of *n* gene loci) computed for 16 different cell types, plotted for 1 ≤ *k* ≤ 200. < *cos* θ*_k_* > provides a measure of the extent of conservation of collective motion shape from 0 (not conserved) to 1 (fully conserved). The curve displays the mean behavior, and the shade, its standard deviation among the 16 different cell types. The bottom panel shows the same plot after reorganizing the order of the modes to select the equivalent modes that best match the reference cell HSPC. (**D**) Example illustrating how mode matching works. In the example, cosine similarity is calculated for each pair of the modes (1 ≤ *k* ≤ 11) from two different cell lines, HSPC (rows) and KBM7 (columns). Linear assignment algorithm is used to find the optimal pairing of modes. (**E**) Similarity of chromosome 17 gene–loci mobility profiles across different cell types after eliminating the differences originating from the frequency dispersion. (**F**) Pearson correlations of the mobility profiles after mode matching. See Zhang *et al*. [51] for more details.

These findings underscore the intricate dependency of 3D chromatin structure on cell type, complicated further by intra-cell type heterogeneity. In this section and the next, we will delve into the dynamic basis of variabilities between different cell types by examining their chromatin mobility profiles inferred from Hi-C data using the GNM by Zhang *et al.* [51]. In that study, we compared the gene loci fluctuation spectra of the entire chromatin for 16 cell types, as predicted by the GNM . The inter-loci contact topology data from public Hi-C datasets [12, 15, 79–82] were used as input. To assess overall similarities in mobility profiles of individual chromosomes across the same cell lines, the MSFs of gene loci were analyzed and compared. As a representative illustration, Figure 4A shows the mobility profiles for chromosome 17 in 16 different cell types, based on the GNM. Cell–cell similarities based on chromosomal mobility profiles were quantified through pairwise Pearson correlation coefficients. This analysis yielded an average Pearson correlation of $r$ = 0.63 ± 0.23 (Figure 4B) for cell–cell mobility profiles of all chromosomes, except for the ectodermal cell line NHEK, which exhibited poor correlation with other cells.

Toward a deeper understanding of the extent of conservation/variation in the fluctuation dynamics of gene loci across different cell types, we generated the mode conservation curve in Figure 4C. The curve represents the average correlation cosine < *cos* θ*_k_* > between the shapes *u_k_*^(*i*)^ and *u_k_*  ^*(j*)^ of mode $k$ accessible to the respective cell types *i* and *j*, averaged over all pairs *(i, j*) of cells, i.e.


(4) \begin{equation*} < {cos}\ \theta_{k} > = {\left[\frac{m\left(m-1\right)}{2}\right]}^{-1}{\sum}_{i=1}^{m-1}{\sum}_{j=i+1}^m\mid{u}_k^{(i)}.{u}_k^{(j)}\mid \end{equation*}

as a measure of the degree of conservation of the collective mode *k* among the *m* cell types, and the shades on the curve indicate the standard deviation for each *k*. Notably, mode 1 (which is the most cooperative mode of collective fluctuations) is closely retained among all 16 cell types, yielding an average correlation of 0.84 ± 0.18. This provides strong support for the prevalence of a closely shared 4D global behavior for chromatin across all cell types, probably consistent with the conservation of the same DNA sequence across the different cell types. The degree of conservation decreased with increasing mode number, with high-to-moderate conservation observed for the most cooperative modes. Further analysis revealed that the modes (indexed from 1 to *n*, in the order of increasing frequency) do not necessarily obey the same order between pairs of cells, i.e. their relative frequencies do not match, as illustrated in panel D for the first 10 modes for HSPC and KBM7 in a heatmap format. The entries in the heatmap represent the mode–mode overlap, varying from 0 (blue) to 1 (red). Swapping of such modes to match the order of the ‘equivalent’ modes between the two cell types enables a higher correlation cosine (bottom plot in panel C), as will be elaborated next.

### Differences in the expressions of modes of motion is a determinant of cell identity

The heat maps in Figure 4D highlight that the diagonal elements do not necessarily show the highest mode–mode overlap, indicating a mismatch in mode numbers between the two cell types. Mode numbers are physically meaningful, with lower indices denoting lower frequency or greater amplitude modes. An off-diagonal red pixel in the heat map signifies shape similarity between two modes but not their frequencies/amplitudes. In other words, a high correlation cosine (or overlap) between the respective *k^th^* and *l*^*t*h^ modes of the cells *i* and *j*, ${u}_k^{(i)}\ \mathrm{and}\ {u}_l^{(j)},$ means the two cells have access to the same mechanism/distribution of fluctuations among the gene loci in their *k^th^* and *l*^*t*h^ modes, but their occurrence frequencies (or respective *λ_k_* and *λ_l_* values) differ. Given that 1/*λ_k_* and 1/*λ_l_* uniformly scale the size of motions, this means the same distribution of motion may be fully effectuated in one cell while it may be suppressed in the other. The variations in mobility profiles of chromosomal loci for chromosome 17 across different cell lines, as illustrated in Figure 4A, can be attributed not only to differences in mode shapes but also to differences in mode frequencies or weights.

To delve further into these distinctions, the mode numbers of HSPCs were employed as a reference, and the modes of the other 15 cell lines were reordered to maximize mode–mode overlaps for chromosome 17 (Figure 4D, bottom panel). This reassignment process retained the shape and frequency of modes but altered their index to align with "equivalent" modes. As a result of this reassignment, the mobility profiles of different cell types became almost indistinguishable across all cell lines (shown for chromosome 17 in Figure 4E). The mode matching thus results in a significant rise in Pearson correlations among the all-versus-all correlations across the diverse cell lines (*r* = 0.85 ± 0.08) depicted in Figure 4F, in comparison to the correlations of 0.63 ± 0.23 observed in Figure 4B. It is noteworthy that some of the equivalent modes had lower weights and might not be as prominently featured in certain cell types.

This analysis suggests that different cell types possess the capacity to undergo similar modes of motions within their chromatin structure, but not all of these modes are put into action, leading to unique distinctions in loci mobility specific to each cell. The differences in the prevalence of "active/expressed" modes among different cell lines appears to be a determinant of cell identity.

### Each cell type has a signature gene mobility profile that correlates with its gene expression profile

The above analysis shows that while different types of cells have access to similar chromosomal dynamics insofar as the MSF profiles of their gene loci in dominant modes are concerned, they exhibit cell-specific variations in the spatial mobility of their genomic loci (see, e.g. Figure 4A) [51]. These differences in spatial dynamics primarily stem from variations in the frequencies of the intrinsically accessible modes of motion between different types of cells. This implies that although different cell types have access to similar collective chromatin movements, they do not necessarily deploy (to the same extent) all available modes of motion.

**Figure 5:**
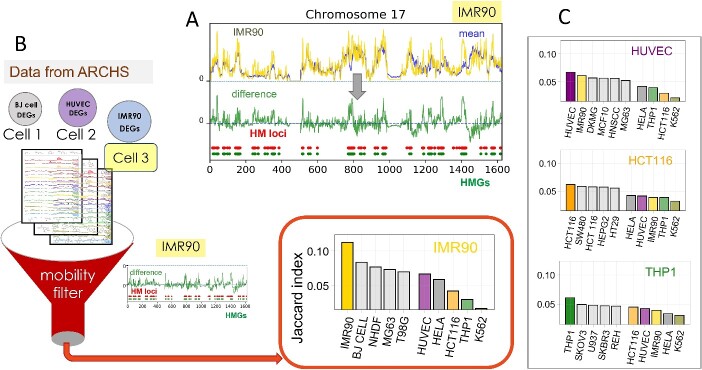
Strong correlation between cell-type-specific HMGs and HEGs. (**A**) Identification of HM loci from the difference (green curve) between the mobility profile obtained with the GNM (for IMR90 chromosome 17; yellow curve)*,* and that averaged across 16 cell lines (blue curve)*;* HM loci are defined as those exhibiting the top 10% mobility, shown by red dots. Genes located in HM loci, HMGs, are shown by green dots. (**B**) The procedure in (**A**) is repeated for all chromosomes; the resulting list of HMGs for IMR90 chromatin is used for screening against the cell-type-specific HEGs compiled in the ARCHS^4^ database. The similarities between the HMGs of the query cell type and the HEGs of the 125 cell types in ARCHS^4^ are quantified by the Jaccard index, and rank-ordered for each query cell type. The bar plot illustrates how IMR90 yields the highest Jaccard index, demonstrating that knowledge of HMGs is sufficient to distinguish cell identity due to strong correlation to the cell-specific HEG profile. (**C**) The procedure repeated for different cell types shows that the queried cell type is extracted as top-ranking cell type (from the pool of 125 in ARCHS^4^) based on high similarity of its HMGs to its HEGs. See more details in Zhang *et al*. [51].

The effective mobility profiles of gene loci within the chromatin may be viewed as a signature property specific to each cell type. This property becomes even more discriminative when we focus on the ‘differences in the mobility profile with respect to the average behavior of different cell types’, illustrated in Figure 5A for lung fibroblasts (IMR90) chromosome 17. This type of difference profile shown in the bottom part of panel A clearly discloses the high mobility (HM) loci as well as the HM genes (HMGs). Similar analysis repeated for all chromosomes yields the complete list of HMGs for the entire chromatin.

The question we asked then is whether the HMGs in a given cell type correlate with the highly expressed genes (HEGs) typical of that cell type. Computations described in Figure 5 indeed showed that the HMG signature is a strongly discriminative feature, which characterizes the cell type by virtue of its strong correlation with the HEGs. Panel B shows that the HMG profile of a specific cell type (e.g. IMR90) screened against the HEG profiles of a series of different types of cells (as documented in the ARCHS^4^ database [83]) accurately detects the cell IMR90 as the cell type whose HEG profile is most similar to the queried HMG profile.

Comparisons with HEGs in multiple cell lines revealed consistent patterns: each query cell line showed the highest similarity to its own HEG pattern among a pool of candidate cell types, as documented in the ARCHS^4^ database [83] (Figure 5C). The similarity metric to compare HMGs and HEGs is the Jaccard index. The Jaccard index measures the overlap between the HMGs of the query cell line and the HEGs of the candidate cell lines, ultimately identifying the top-ranking candidate cell lines HEG pattern (as a function of gene locus) is most similar to the HMG pattern of the query cell line. Notably, the top-ranking candidate cell line consistently matched the query cell line itself in all cases, providing a compelling evidence into the relationships between gene mobility and its expression.

## CONCLUSION

Recent advances in experimental and computational technologies are now shedding light to the 4D behavior of the chromosomes, defined by their 3D structure and dynamics. The 3D properties of the genome are probably best described by structural models compatible with well-defined topology of contacts between gene loci. The 3D topology influences the accessibility of genes and regulatory elements, impacting the precise timing and extent of gene expression. Moreover, the 3D contact topology across gene loci plays a pivotal role in orchestrating gene–gene couplings, evolutionary adaptation and cellular functions by ensuring that genes are activated or repressed in the right context, at the right time and in response to various signals to maintain homeostasis [84, 85]. In this context, it became increasingly important to understand, not only the 3D but also 4D genome, which incorporates the time dependency. 4D properties encompass structural changes over a wide range of time spans, from microscopic thermally driven fluctuations—which is the focus of the current study, to changes accompanying development and diseases, to evolutionary adaptations.

The GNM has proven to be a powerful analytical approach adaptable to structural or contact data at multiple scales, and the present review emphasizes its utility for exploring the 4D properties of the chromatin, when used in conjunction with Hi-C data. Its suitability stems from its ability to take account of the overall coupling/connectivity between vast numbers of structural elements, in this case gene loci. Human chromosomes span millions of base pairs, resulting in thousands of bins per chromosome at a 5 kb resolution, and the analysis may be performed for the entire chromatin even taking account of weak but existing inter-chromosomal couplings. GNM's scalability and adaptability make it remarkably well suited for characterizing chromatin dynamics under equilibrium conditions, and its robustness allows to predict reproducible (global) features such as the signature mobility profiles of the chromosomes, even when using as input noisy or imprecise Hi-C data.

Here is a summary of what we learned about chromatin structure and dynamics using the GNM. The first application in 2017 [49] showed us that gene loci accessibilities measured ATAC-seq and DNase-seq for GM12878 and IMR90 could be satisfactorily explained by the GNM, as well as ChIP-seq data on gene–gene correlations. The level of agreement between theory and experiments, despite the simplicity of the theory, taught us that inter-loci contact topology, which is the major ingredient of the GNM, is a major determinant of gene loci accessibility in those experiments. We also noted that DNase-seq data yielded higher agreement (than ATAC-seq) with the GNM, which brought up the possibility that DNase-seq measurements might be more accurate. Note that GNM can readily generate such data (on MSFs of gene loci and correlations between distal loci) not directly observable by experiments, and the good agreement with those data observed so far in experiments gives us confidence about the possible use of the GNM for further learning about such properties (in the absence of experimental data). Secondly, by systematically comparing GNM modes across 16 human cell lines, we learned that different types of cells have all access to a very similar repertoire of global modes, yielding an average correlation of 0.84 between the shape of their global modes. However, their MSF profiles yields a correlation of 0.63 ± 0.23. The origin of this discrepancy lies in the different prior probabilities/frequencies of this existing pool of modes, some modes being more readily accessible than others in specific types of cells. Thus, in the same way as the same DNA sequence exists but not the same levels of proteins are expressed in different cells, we also have a well-defined pool of available motions, but not all of them are being deployed. The GNM identified this important relation—it is not about pre-existing modes of motion, but the ‘recruitment’ of these modes, which differentiates the different types of cells. Evidently, this selection of particular modes is a manifestation of evolutionary pressure to perform the specific cell type function. We also learned that the mobility profiles of the gene loci, as predicted by the GNM, can be used as a ‘filter’ for recognizing the type of the cell, upon comparison with gene-expression profiles in public databases. Each cell type has a unique gene loci mobility profile, which uniquely relates to cell-specific gene expression profiles (Figure 5). These findings underscore the crucial role of chromatin dynamics in the predisposition of the genome to lineage-specific gene expression during differentiation.

Further advances in computer-aided characterization of chromatin structure and dynamics research offer promising avenues for advancing our understanding of genome function. One promising avenue is use of the anisotropic network model (ANM) [86, 87] for analyzing the data compiled in the Genome Structure Database (GSDB) [88]. In contrast to the GNM, which requires contact information exclusively (and is thus ideally suited for inferring genome structural dynamics from Hi-C data without dependency on structural models), ANM uses 3D coordinates and predicted *3n*-dimensional vectors for the collective movements of a network of *n* nodes in 3D space. GSDB contains tens of thousands of 3D chromatin structural models generated by applying different 3D reconstruction methods to various Hi-C datasets. ANM analysis of these structural models can provide insights into recurrent dynamic features of the chromosomes or entire chromatin by providing animations of the collective movements of the gene loci themselves in 3D (not only their size or correlations), thus elucidating how structural changes within the chromatin may relate to expression and regulation. Yet, we anticipate that the MSFs and cross-correlations predicted by the GNM would be more accurate than those predicted by the ANM, as GNM predictions depend on contact data directly, while ANM has another layer of approximation—the structural models derived from contact data.

Finally, the GNM is a model purely based on geometry (in this case, inter-loci contact topology). As such, it predicts the collective dynamics driven by entropic effects, as has been also discussed in the original work of Flory and coworkers [18] and our initial studies [47, 54]. It does not contain any (other) energetic contributions to the stability or kinetics of the examined structure. The reason it works is probably the fact that it provides a rigorous mathematical description of the intricate contact topology, incorporating the details on the connectivity/interactions of thousands or even millions of nodes. The underlying assumption of nodes and springs obeying Gaussian dynamics is exact for infinitely large systems (due to central limit theorem), and in this respect, the chromatin appears to be an even more suitable system to be modeled by the GNM than much smaller proteins to which it has been widely applied. However, we recognize that the 4D behavior of the genome is affected by several factors not included here. For example, investigating the presence and role of histone modification markers at domain boundaries [27] using ENM-based analysis holds potential to uncover regulatory mechanisms governing gene expression. No effects of cohesion on loop domains [15], or no specific mechanisms such as loop extrusion are accounted for by the GNM. Our previous work also demonstrated the critical role of post-translational modifications including methylation and ubiquitylation in genome stability [89] and carcinogenesis. The GNM simply provides an overall view of the structural dynamics intrinsically accessible to the genome, including correlations within and across the chromosomes and gene loci pairs separated by 100s of Mbs, but its adoption to modeling more and more localized events that depend on specific interactions is not possible without proper rescaling of model parameters and/or adopting multiscale hybrid methods [90]. Given that the GNM is also applicable at the molecular level (of proteins, DNA and their complexes, including nucleosomes [91]), it is conceivable that the model could be adopted to exploring the mesoscale between the overall chromatin and individual nucleosomes. These future directions are expected to collectively contribute to unraveling the intricate relation between chromatin structural dynamics and gene expression and regulation as a function of cell type.

**Key Points**

- Advances in computational modeling of complex systems with elastic network models (ENMs) provide new insights into chromatin 4D properties.
- The use of Hi-C data as input in the Gaussian network model (GNM) enables physics-based evaluation of chromatin accessibility and gene loci cross-correlations.
- Differential mobilities and solvent exposures of distinct genomic compartments and subcompartments are captured by ENMs.
- Hi-C data from different cells used in the GNM permit us to characterize cell-specific gene mobility profiles.
- Gene mobility profiles serve as signature properties that correlate with the cell-specific differential expressions of genes.

## Author Biographies

**Anupam Banerjee** is a senior postdoctoral associate working on the integration of multiscale physics-based models and machine-learning methods toward a comprehensive understanding of biomolecular structure–dynamics–function relationships.

**She Zhang** is a scientific developer presently working on enhanced sampling methods to estimate drug-like molecules’ permeability and developing tools for structure-based simulations. He has extensive experience in chromosomal dynamics modeling using Hi-C contact data.

**Ivet Bahar** is a computational and systems biologist and an internationally renowned scientist known for pioneering novel models and methods in structural, computational and molecular biophysics, including the widely used elastic network models for protein dynamics.
